# Impact of polycystic kidney disease on outcomes after renal transplantation: systematic review and meta-analysis

**DOI:** 10.1080/0886022X.2025.2611618

**Published:** 2026-01-19

**Authors:** Chao Liu, Fu Yan, Minghua Zhong, Yulin Niu

**Affiliations:** Department of Organ Transplantation, Affiliated Hospital of Guizhou Medical University, Guiyang, Guizhou Province, China

**Keywords:** Polycystic kidney disease, renal transplantation, patient survival, graft survival, posttransplant diabetes mellitus

## Abstract

Polycystic kidney disease (PKD) represents one of the most prevalent inherited renal disorders and constitutes the fourth leading etiology of end-stage kidney disease (ESKD). While renal transplantation remains the optimal therapeutic intervention for ESKD, existing evidence regarding post-transplant survival rates and graft loss in PKD recipients remains contentious. This systematic review incorporated studies from PubMed, Scopus, and Embase databases, with a preregistered protocol on PROSPERO (CRD420251002570). We evaluated the impact of PKD on post-transplant outcomes through comparative analyses of patient survival and graft survival at 1-, 5-, and 10-year intervals between PKD and non-PKD cohorts. Secondary outcomes included complication profiles. Methodological quality was appraised using the ROBINS-I tool for non-randomized studies. From 1,187 screened records, 23 studies were eligible for inclusion. Meta-analysis demonstrated superior 1-year patient survival in PKD kidney transplant recipients (OR 1.30, 95% CI: 1.08–1.56; 19 studies, I^2^=0.0%) and enhanced 10-year graft survival (OR 1.60, 95% CI: 1.52–1.62; 20 studies, I^2^=73.5%) compared to non-PKD counterparts. Subgroup analyses revealed amplified survival advantages in retrospective studies (OR 1.67, 95% CI: 1.35–2.07; 7 studies, I^2^=23.6%) and cohorts with unspecified donor types (OR 1.27, 95% CI: 1.05–1.53; 13 studies, I^2^=0.0%). Notably, PKD recipients exhibited higher incidence of post-transplant diabetes mellitus (OR 1.61, 95% CI: 1.40–1.81; I^2^=85.4%), while acute rejection episodes, infectious complications, and malignancy rates showed no intergroup divergence. PKD kidney transplant recipients demonstrate favorable short-term survival and long-term graft retention compared to non-PKD patients. However, residual confounding from donor characteristics, sample size heterogeneity, and methodological variations necessitate cautious interpretation.

## 1. Introduction

Polycystic kidney disease (PKD), one of the most prevalent monogenic renal disorders, affects over 12 million individuals globally, imposing substantial public health burdens [1,2]. This condition is pathologically defined by progressive cystogenesis, leading to nephromegaly, infected cysts, nephrolithiasis, and progressive deterioration of renal function and quality of life [3,4]. In the United States, PKD accounts for 11–12 cases of end-stage kidney disease (ESKD) per million population annually, ranking as the fourth most common etiology of ESKD [5]. Despite emerging therapeutic strategies targeting PKD pathogenesis [6,7], kidney transplantation remains the preferred treatment modality for PKD patients progressing to ESKD [8,9]. However, clinical challenges persist due to native nephromegaly, hepatic cystic involvement, and cardiovascular comorbidities, which may adversely impact transplant surgical outcomes and long-term prognosis [3,10,11].

Contemporary advancements in immunosuppressive regimens and surgical techniques have not fully resolved controversies surrounding post-transplant outcomes in PKD populations. While some cohort studies suggest superior graft survival and patient survival in PKD recipients compared to non-PKD ESKD counterparts [9,12,13], conflicting evidence highlights elevated risks of acute rejection, graft failure, and mortality, alongside increased incidence of post-transplant diabetes mellitus (PTDM) and malignancies [14,15]. Notably, a comprehensive synthesis of global evidence through meta-analytic approaches remains conspicuously absent.

This meta-analysis systematically evaluates the impact of PKD on post-transplant survival and graft retention outcomes, aiming to provide transplant clinicians with evidence-based insights for pre-transplant risk stratification and post-operative management.

## 2. Methods

The pre-specified protocol for this meta-analysis was registered with PROSPERO (ID: CRD420251002570) and conducted in accordance with PRISMA (Preferred Reporting Items for Systematic Reviews and Meta-Analyses) guidelines [16].

### 2.1. Literature search

A systematic search of PubMed, Scopus, and Embase was performed for studies published between January 2000 and February 2025. To mitigate single-investigator bias, dual independent screening was conducted by two researchers using a predefined search strategy combining controlled vocabulary and free-text terms: ‘Polycystic Kidney Diseases’, ‘Autosomal Dominant Polycystic Kidney Disease’, ‘Polycystic Kidney’, ‘Renal Transplantation’, ‘Kidney Transplant’, ‘Graft Survival’, ‘Patient Survival’, ‘Overall Survival’ and ‘Transplant Outcomes’. Boolean operators (AND/OR) were applied to optimize sensitivity/specificity (full strategy in Supplementary Table 1). After deduplication, titles/abstracts were screened for relevance, followed by full-text eligibility adjudication. Discrepancies were resolved through consensus with a third investigator. Reference lists of included studies were hand-searched to identify additional records.

### 2.2. Inclusion/exclusion criteria

Inclusion criteria: (a) Cohort studies (retrospective/prospective), randomized controlled trials, or case-control designs enrolling adult kidney transplant recipients (>18 years). (b) Direct comparison between ESKD patients with PKD and non-PKD etiologies. (c) Reporting of ≥1 primary outcome: patient survival/mortality or graft survival/loss. (d) Minimum follow-up duration of 1-year post-transplantation.

Exclusion criteria: (a) Studies involving repeat transplantation recipients. (b) Non-primary research (conference proceedings, reviews, case reports, editorials). (c) Insufficient outcome reporting. (d) Non-English publications. (e) Duplicate cohorts (for overlapping datasets, the most comprehensive study was retained).

### 2.3. Data extraction

Two investigators independently extracted the following variables using standardized forms: (a) Study Characteristics: first author, publication year, geographic region; (b) Demographic Data: recipient age at transplantation and proportion of female participants; (c) Clinical Parameters: follow-up duration, graft type (deceased or living donor), and etiology of ESKD; (d) Therapeutic Variables: immunosuppressive regimens, including induction and maintenance protocols; (e) Primary Outcomes: patient survival (all-cause mortality); (f) Secondary Outcomes: graft survival or loss, PTDM, acute rejection, infectious complications, and *de novo* malignancies. Discrepancies were resolved through iterative review and consultation with a senior investigator.

### 2.4. Quality assessment

Methodological rigor of observational studies was evaluated using the Risk of Bias In Non-randomized Studies of Interventions (ROBINS-I) tool [17,18]. Two trained investigators (Fu Yan and Minghua Zhong) independently assessed seven bias domains: Confounding, Participant selection, Intervention classification, Deviations from intended interventions, Missing data, Outcome measurement and Selective reporting. Studies were categorized as low/moderate/serious/critical risk through consensus adjudication. Publication bias was evaluated *via* funnel plot asymmetry and quantitative Egger’s regression test [19].

### 2.5. Statistical analysis

All statistical analyses were performed using R software (R 4.4.1). Differences were considered statistically significant when *p* < 0.05. The combined effect sizes were reported as odds ratios (OR) with 95% confidence intervals (CI). Heterogeneity was categorized based on the I^2^ statistic, with low (I^2^ < 25%), moderate (25% < I^2^ < 50%), and high (I^2^ > 75%) levels of heterogeneity. A fixed-effect model was chosen when heterogeneity was low, while a random-effects model was used when heterogeneity was high. Additionally, subgroup analyses were conducted based on donor type (deceased donors and living donors), study design, and sample size (≤200 and >200). Sensitivity analyses were performed to assess the stability of the combined results by excluding each individual study.

## 3. Results

### 3.1. Study characteristics

**Figure 1.**
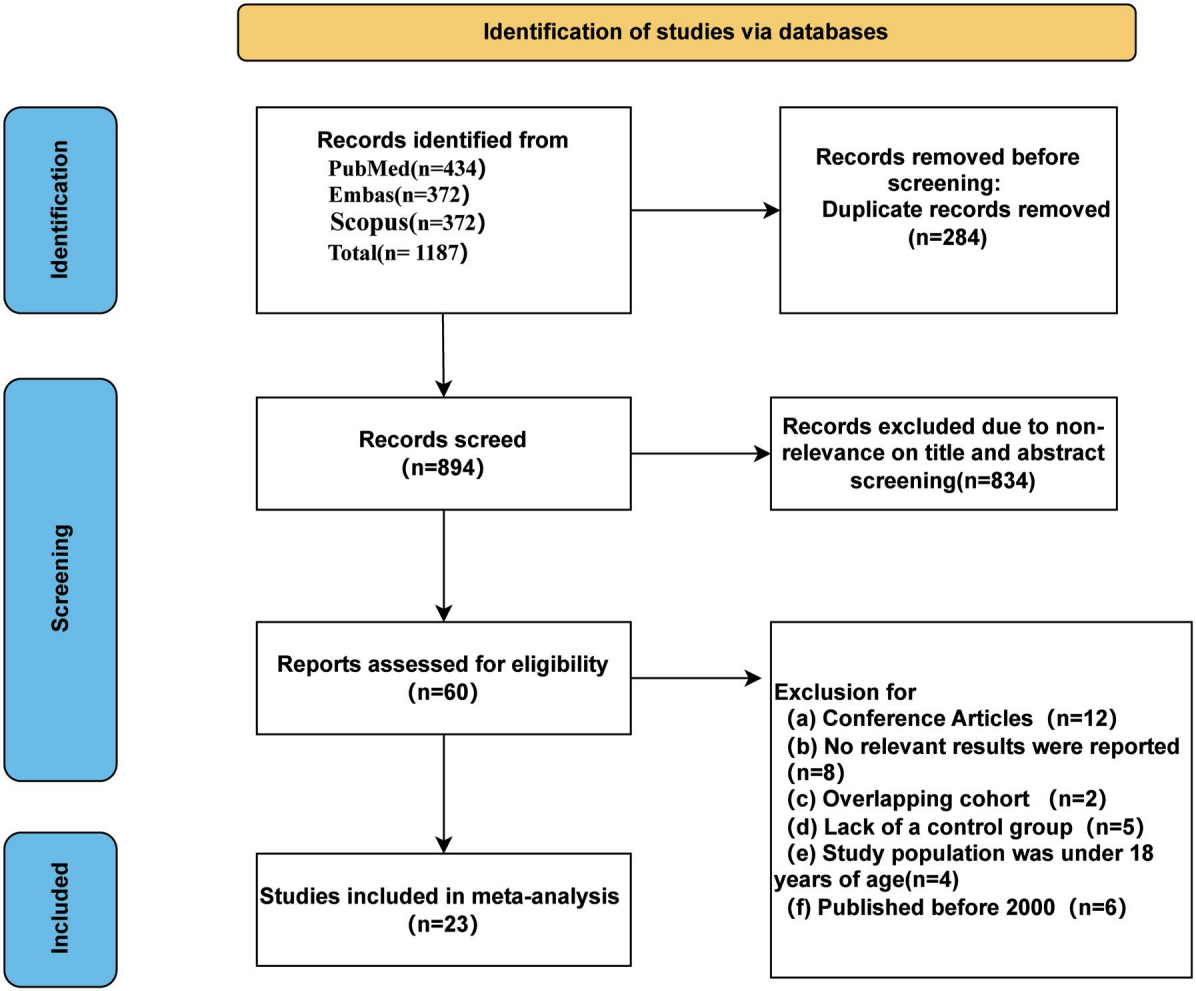
Flow chart for PRISMA 2020 study selection.

The study selection process is delineated in Figure 1. Systematic screening of PubMed, Embase, and Scopus yielded 1,187 records. After removing 293 duplicates, 894 studies underwent title/abstract screening, with 834 excluded for irrelevance. Full-text review of 60 potentially eligible studies culminated in 23 meeting inclusion criteria for quantitative synthesis.

Key characteristics of included studies are summarized in Table 1. Among the 23 studies, 7 employed case-control designs and 16 were retrospective cohort analyses. Geographically, 11 studies originated from European centers, 7 from Asian institutions, 2 each from North and South America, and 1 from Africa. Two multicenter investigations spanned multiple regions. Female representation ranged from 30 to 40% across most cohorts, with Waiser et al. [9] reporting disproportionately higher female enrollment (see Table 1). Donor stratification revealed:11 studies included both deceased and living donors, 2 studies exclusively used living donors and 9 studies lacked donor source specification. Non-PKD ESKD etiologies comprised IgA nephropathy (24.1%), lupus nephritis (18.5%), diabetic nephropathy (15.3%), primary glomerulopathies (12.7%), hereditary/familial nephropathies (9.8%), ANCA-associated vasculitis (8.1%), and membranous nephropathy (6.2%). The PKD cohorts encompassed 13,845 recipients (sample range: 18-7,181), compared to 41,360 non-PKD controls (range: 18-11,150). Mean recipient age at transplantation varied from 36.1 to 57.2 years across studies.

**Table 1.** Basic characteristics of the patients receiving kidney transplantation.

Study	Study design	Country	Age	Female (%)	Causes of cORonic renal failure(control)	Immunosuppressive regimens	Type of donor	PKD	Non-PKD
Waiser [9]	Case-control	Germany	55.1	81	Diabetic nepORopathy, Vascular nepORopathy, Primary glomerular disease, Hereditary nepORopathy, CORonic pyelonepORitis, Others	no significant difference	LD accounted for 66%	189	189
Xie [33]	Retrospective cohort	China	37.6	41	IgA nepORopathy, Lupus nepORitis, Focal segmental glomerulosclerosis (FSGS), Antineutrophil cytoplasmic antibody (ANCA)-associated vasculitis, Membranous nepORopathy, Others	NA	DD accounted for 65%	148	550
Rothem [24]	Retrospective cohort	Israel	54.9	29.6	NA	NA	LD accounted for 78%	81	432
Lee [29]	Case-control	Korea	47	40.8	Diabetic nepORopathy, Hypertensive nepORopathy, GlomerulonepORitis, Others	no significant difference	LD accounted for 68%	189	4263
Garland [26]	Retrospective cohort	Britain	47.5	37.2	GlomerulonepORitis	NA	DD accounted for 83%	126	266
Schellekens [12]	Retrospective cohort	Belgium	55.5	39	GlomerulonepORitis, Diabetic nepORopathy, Hypertensive nepORopathy, Familial nepORopathy, Others	no significant difference	NA	392	1698
Tsai [14]	Retrospective cohort	China	42.9	45.2	NA	NA	NA	41	1102
Chedid [25]	Case-control	America	57.2	40.6	Hypertensive nepORopathy, GlomerulonepORitis, Others	NA	NA	271	271
Bhutani [13]	Retrospective cohort	America	49.2	39.8	CORonic glomerulonepORitis, Hypertensive nepORopathy, IgA nepORopathy, Diabetic nepORopathy, Unknown etiology	no significant difference	DD accounted for 60%	619	4312
Li [32]	Case-control	China	47.2	35.3	CORonic glomerulonepORitis, Hypertensive nepORopathy, IgA nepORopathy, Diabetic nepORopathy, Unknown etiology	no significant difference	NA	68	68
Favi [34]	Retrospective cohort	Italy	44.6	36.1	Immune-mediated kidney disease	NA	NA	67	99
Pruthi [48]	Retrospective cohort	Britain	47.9	37.8	IgA nepORopathy	NA	DD accounted for 69%	1775	1069
Pippias [15]	Retrospective cohort	Netherlands	51.2	37.8	GlomerulonepORitis, Crescentic (extracapillary) glomerulonepORitis, GlomerulonepORitis, histological examination not provided above	NA	DD accounted for 78%	7181	8056
Nieto-Ríos [49]	Retrospective cohort	Columbia	41.1	63.8	Lupus nepORitis	significant difference	NA	31	27
Mashaly [50]	Retrospective cohort	Egypt	36.1	29.5	Hypertensive nepORosclerosis	significant difference	LD accounted for 100%	55	57
Mosconi [51]	Retrospective cohort	Italy	48	35.3	NA	NA	NA	1709	11150
Vega [52]	Case-control	Chile	46	26.3	CORonic glomerulonepORitis, CORonic pyelonepORitis, NepORosclerosis, Kidney disease of unknown cause	no significant difference	DD accounted for 73.3%	19	38
Kute [53]	Case-control	India	48	17.8	NA	no significant difference	DD accounted for 100%	18	18
Jacquet [54]	Retrospective cohort	France	45.15	37.8	NA	no significant difference	DD accounted for 99%	543	4779
Gonçalves [55]	Retrospective cohort	Portugal	44.7	33.9	NA	no significant difference	DD accounted for 90%	48	397
Johnston [56]	Retrospective cohort	Eire	42.6	35.2	Non-diabetic kidney disease	NA	NA	176	1185
Stiasny [57]	Case-control	Germany	NA	NA	NA	NA	NA	80	88
Shiroyanagi [58]	Retrospective cohort	Japan	NA	NA	NA	NA	LD accounted for 100%	19	1246

Abbreviations: PKD, polycystic kidney disease; DD, Donor of death; LD, living donor; NA, not improved.

### 3.2. Patient survival

**Figure 2.**
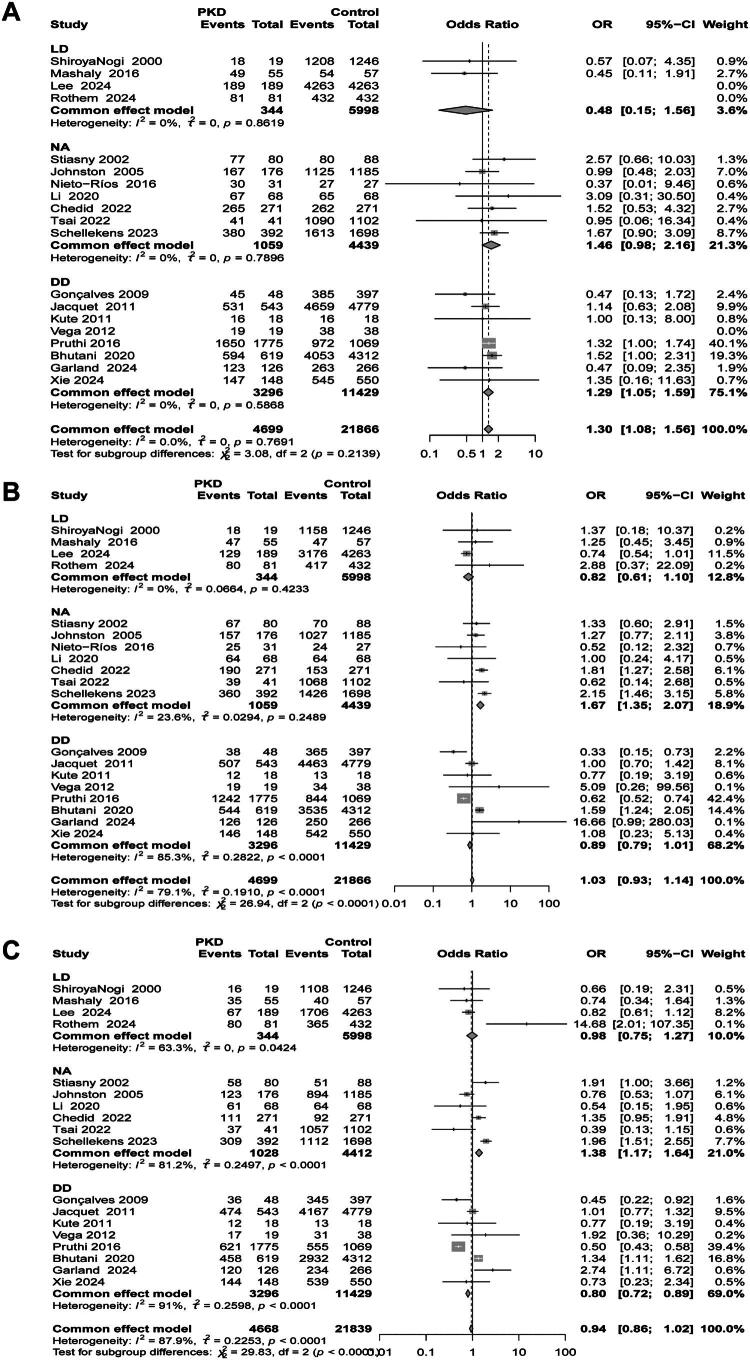
Forest plot of Patient survival in the PKD group and the non-PKD group. (a) 1-year survival rates; (b) 5-year survival rates; (c) 10-year survival rate.

As depicted in Figure 2, PKD recipients demonstrated significantly superior 1-year survival compared to non-PKD controls (OR 1.30, 95% CI: 1.08–1.56; 19 studies, I^2^=0.0%). However, this survival advantage attenuated over longer follow-up intervals, with no significant intergroup differences observed at 5-years (OR 1.03, 95% CI: 0.93–1.14; I^2^=79.1%) or 10-years (OR 0.94, 95% CI: 0.86–1.02; I^2^=87.9%). Funnel plot symmetry (Supplementary Figure S1) and Egger’s regression (1-year: *p* = 0.59; 5-year: *p* = 0.55; 10-year: *p* = 0.79) indicated no publication bias. Sensitivity analyses confirmed robustness, with no material changes upon sequential study exclusion (Supplementary Figure S2). In the subgroup analysis based on donor type, the 1-year survival rate analysis showed that the survival rate of PKD patients in the living donor group was lower than that of the non-PKD group, although this difference was not statistically significant (OR 0.48, 95% CI: 0.15, 1.56; *N* = 4, I^2^ = 0.0%). In contrast, the results for the deceased donor group were consistent with the overall study findings (OR 1.26, 95% CI: 1.05, 1.59; *N* = 8, I^2^ = 0.0%). Furthermore, the subgroup analysis of 5-year and 10-year survival rates indicated that PKD patients from the donor group with unspecified sources had significantly better survival rates compared to non-PKD patients (5-year: OR 1.67, 95% CI: 1.35, 2.07; *N* = 7, I^2^ = 23.6%; 10-year: OR 1.38, 95% CI: 1.17, 1.64; *N* = 6, I^2^ = 81.2%). However, the survival rate results for both the living donor group and the deceased donor group did not reach statistical significance. Common causes of death in recipients include infections, cardiovascular events, and malignancies.

In the subgroup analysis based on sample size, studies with a larger sample size (*N* > 200) were compared to those with a smaller sample size (*N* ≤ 200). The analysis of the 1-year survival rate showed that PKD patients in studies with a larger sample size had significantly better survival rates (OR 1.31, 95% CI: 1.08, 1.57; *N* = 13, I^2^ = 0.0%), while studies with a smaller sample size did not show a significant association (OR 1.19, 95% CI: 0.57, 2.51; *N* = 6, I^2^ = 3.4%). In the analysis of 5-year and 10-year survival rates, no significant associations were observed regardless of sample size (see Table 2, Supplementary Figure S3). Additionally, the subgroup analysis based on study design indicated that in retrospective cohort studies, the survival rate of PKD patients was significantly better than that of non-PKD patients for the 1-year survival rate (OR 1.27, 95% CI: 1.05, 1.53; *N* = 13, I^2^ = 0.0%). On the other hand, when the results of case-control studies were combined, the survival rate for the PKD group did not reach statistical significance (OR 1.83, 95% CI: 0.89, 3.77; *N* = 6, I^2^ = 0.0%). Similarly, the subgroup analysis of 5-year and 10-year survival rates based on study design showed results consistent with the sample size subgroup analysis, with no statistical significance observed (see Table 2, Supplementary Figure S4).

**Table 2.** Subgroup analysis.

	Patient survival	Graft survival
Sample		
*N* > 200		
One year	OR 1.31 (95% CI: 1.08, 1.57) (*N* = 13, I² = 0.0%,*)	OR 0.99 (95% CI: 0.91, 1.07) (*N* = 14, I² = 22.5%)
Five years	OR 1.02 (95% CI: 0.92, 1.14) (*N* = 13, I² = 85.6%)	OR 1.00 (95% CI: 0.95, 1.06) (*N* = 14, I² = 86%)
Ten years	OR 0.9 3(95% CI: 0.86, 1.01) (*N* = 13, I² = 91%)	OR 1.61 (95% CI: 1.53, 1.69) (*N* = 14, I² =80.1%,*)
*N* ≤ 200		
One year	OR 1.19 (95% CI: 0.58, 2.51) (*N* = 6, I² = 3.4%)	OR 1.21 (95% CI: 0.72, 2.05) (*N* = 7, I² = 0%)
Five years	OR 1.14 (95% CI: 0.70, 1.84) (*N* = 6, I² = 0%)	OR 1.37 (95% CI: 0.93, 2.02) (*N* = 7, I² = 0%)
Ten years	OR 1.16 (95% CI: 0.76, 1.76) (*N* = 5, I² = 27.5%)	OR 1.41 (95% CI: 1.01, 1.99) (*N* = 6, I² = 17.3%,*)
Study design		
Retrospective cohort		
One year	OR 1.27 (95% CI: 1.05, 1.53) (*N* = 13, I² = 0%,*)	OR 0.99 (95% CI: 0.91, 1.08) (*N* = 15, I² = 19.2%)
Five years	OR 1.00 (95% CI: 0.90, 1.13) (*N* = 13, I² = 82.6%)	OR 1.01 (95% CI: 0.96, 1.07) (*N* = 15, I² = 85%)
Ten years	OR 0.92 (95% CI: 0.84, 1.00) (*N* = 12, I² = 91.4%)	OR 1.64 (95% CI: 1.55, 1.72) (*N* = 15, I² = 74.3%,*)
Case-control		
One year	OR 1.83 (95% CI: 0.89, 3.77) (*N* = 6, I² = 0%)	OR 1.18 (95% CI: 0.67, 2.07) (*N* = 6, I² = 0%)
Five years	OR 1.13 (95% CI: 0.90, 1.41) (*N* = 6, I² = 67.5)	OR 0.93 (95% CI: 0.74, 1.16) (*N* = 6, I² = 0%)
Ten years	OR 1.07 (95% CI: 0.87, 1.32) (*N* = 6, I² = 46.7%)	OR 1.08 (95% CI: 0.86, 1.35) (*N* = 6, I² = 44.3%)

*Statistically significant at *p* < 0.05.

Subgroup analysis stratified by age (>45 years vs. ≤45 years) (Table 3) showed no statistically significant differences in survival rates at 1 year, 5 years, and 10 years between the PKD group and the non-PKD group, regardless of whether in the smaller age subgroup (≤45 years) or the larger age subgroup (>45 years). Additionally, subgroup analysis based on dialysis duration (>24 months vs. ≤24 months) was performed (Table 3). The results showed that among patients with shorter dialysis duration (≤24 months), the PKD group had superior survival rates at 5 years and 10 years compared to the non-PKD group (5-year survival: OR 1.34, 95% CI: 1.13–1.58, *N* = 7, I^2^=74.7%; 10-year survival: OR 1.21, 95% CI: 1.05–1.40, *N* = 6, I^2^=60.5%). However, there was no statistically significant difference in 1-year survival between the two groups. Conversely, among patients with longer dialysis duration (>24 months), the PKD group had significantly worse survival rates at 5 years and 10 years than the non-PKD group (5-year survival: OR 1.34, 95% CI: 1.13–0.84, *N* = 6, I^2^=86.2%; 10-year survival: OR 0.79, 95% CI: 0.70–0.88, *N* = 6, I^2^=94.1%).

**Table 3.** Subgroup analysis by age and duration of dialysis.

	Patient survival	Graft survival
Age (years)		
>45		
One year	OR 1.37 (95% CI: 1.13, 1.67) (*N* = 11, I² = 0.0%)	OR 0.99 (95% CI: 0.91, 1.08) (*N* = 13, I² = 23.4%)
Five years	OR 1.03 (95% CI: 0.93, 1.15) (*N* = 11, I² = 79.1%)	OR 1.00 (95% CI: 0.95, 1.05) (*N* = 13, I² = 86%)
Ten years	OR 0.96 (95% CI: 0.88, 1.05) (*N* = 11, I² = 92.2%)	OR 1.63 (95% CI: 1.54, 1.71) (*N* = 13, I² =77.7%, *)
≤45		
One year	OR 0.78 (95% CI: 0.46, 1.32) (*N* = 6, I² = 0%)	OR 1.07 (95% CI: 0.74, 1.56) (*N* = 6, I² = 0%)
Five years	OR 0.92 (95% CI: 0.65, 1.31) (*N* = 6, I² = 45.3%)	OR 1.30 (95% CI: 0.99, 1.71) (*N* = 6, I² = 34.5%)
Ten years	OR 0.68 (95% CI: 0.52, 0.90) (*N* = 5, I² = 0%)	OR 1.21 (95% CI: 0.95, 1.55) (*N* = 5, I² = 41.1%)
Dialysis time (months)		
>24		
One year	OR 1.33 (95% CI: 1.06, 1.67) (*N* = 6, I² = 0%)	OR 1.03 (95% CI: 0.84, 1.25) (*N* = 7, I² = 0%)
Five years	OR 0.84 (95% CI: 0.73, 0.97) (*N* = 6, I² = 86.2%,*)	OR 1.43 (95% CI: 1.21, 1.66) (*N* = 7, I² = 0%)
Ten years	OR 0.79(95% CI: 0.70, 0.88) (*N* = 6, I² = 94.1%,*)	OR 1.89 (95% CI: 1.68, 2.12) (*N* = 7, I² = 73.5%, *)
≤24		
One year	OR 1.42 (95% CI: 0.99, 2.04) (*N* = 7, I² = 0%)	OR 0.93 (95% CI: 0.83, 1.04) (*N* = 7, I² = 27.9%)
Five years	OR 1.34 (95% CI: 1.13, 1.58) (*N* = 7, I² = 74.7%,*)	OR 0.88 (95% CI: 0.83, 0.94) (*N* = 7, I² = 80.5%, *)
Ten years	OR 1.21 (95% CI: 1.05, 1.40) (*N* = 6, I² = 60.5%,*)	OR 1.47 (95% CI: 1.37, 1.57) (*N* = 6, I² = 66.3%, *)

*Statistically significant at *p* < 0.05.

### 3.3. Graft survival

**Figure 3.**
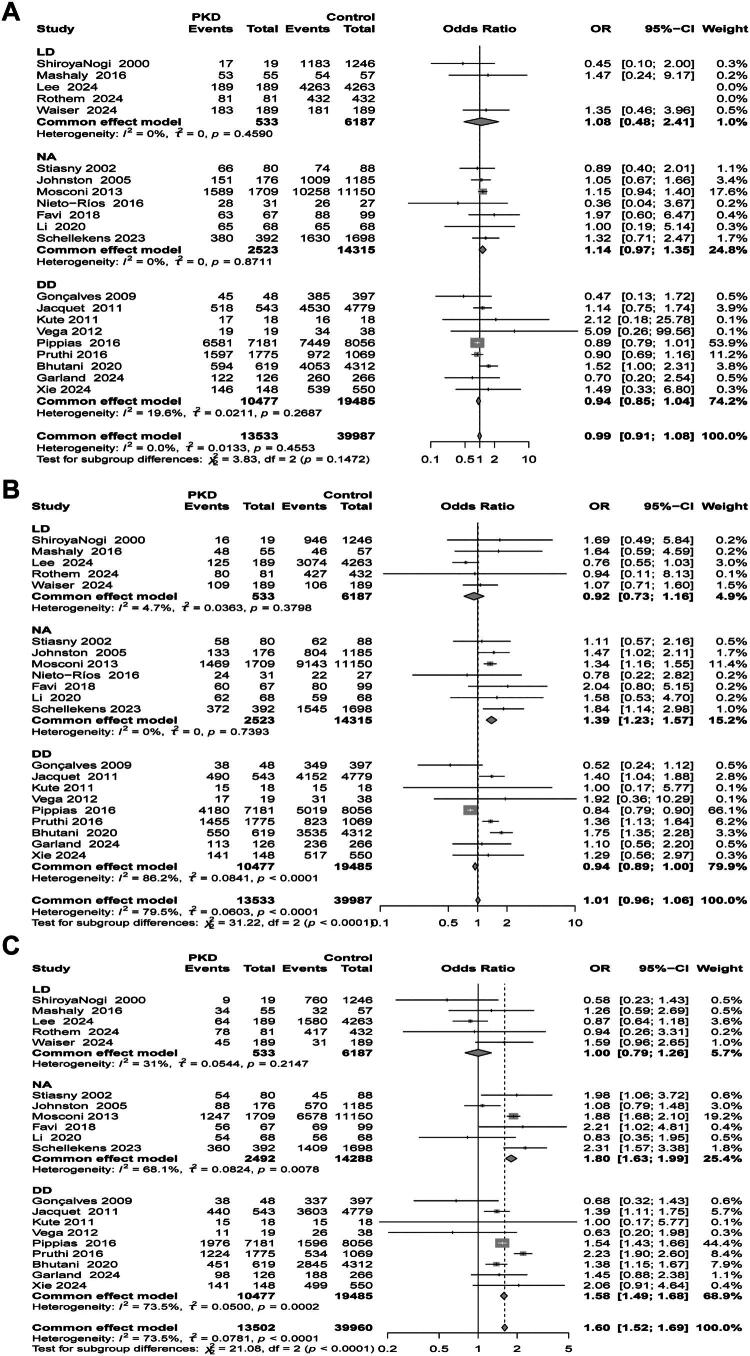
Forest plot of graft survival in the PKD group versus the non-PKD group. (a) 1-year graft survival rate; (b) 5-year graft survival rate; (c) 10-year graft survival rate.

The results are shown in Figure 3. There were no significant differences in graft survival rates between the PKD group and the non-PKD group at both 1-year and 5-years (1-year: OR 0.99, 95% CI: 0.91, 1.08; *N* = 21, I^2^ = 0.0%; 5-years: OR 1.01, 95% CI: 0.96, 1.06; *N* = 22, I^2^ = 79.5%). Notably, the analysis of the 10-year graft survival rate revealed that the PKD group had significantly better graft survival compared to the non-PKD group (OR 1.60, 95% CI: 1.52, 1.62; *N* = 20, I^2^ = 73.5%). Additionally, the results of the Egger test (1 year: *p* = 0.80; 5 years: *p* = 0.07; 10 years: *p* = 0.60) and the funnel plot (Supplementary Figure S5) indicated no evidence of publication bias. The sensitivity analysis demonstrated stability, with no change in significance after excluding any individual study (Supplementary Figure S6). In the subgroup analysis based on donor type, the analysis of the 10-year graft survival rate showed that PKD patients in the deceased donor group had significantly higher graft survival compared to the non-PKD group (OR 1.58, 95% CI: 1.49, 1.68; *N* = 20, I^2^ = 73.5%). However, in the living donor group, there was no significant difference in graft survival between the PKD and non-PKD groups (OR 1.58, 95% CI: 1.49, 1.68; *N* = 20, I^2^ = 73.5%). Furthermore, in the subgroup analysis of the 5-year graft survival rate, PKD patients in the unspecified donor source group had significantly better graft survival than the non-PKD group (OR 1.39, 95% CI: 1.23, 1.57; *N* = 7, I^2^ = 0%), while neither the living donor group nor the deceased donor group showed statistical significance (living donor group: OR 0.92, 95% CI: 0.73, 1.16; *N* = 5, I^2^ = 74.7%; deceased donor group: OR 0.94, 95% CI: 0.89, 1.00; *N* = 9, I^2^ = 86.2%). Common reasons for recipient graft loss included acute rejection, chronic rejection, BK virus-associated nephropathy, and recurrent chronic glomerulonephritis.

Additionally, subgroup analyses were conducted based on sample size, comparing studies with a large sample size (*N* > 200) to those with a small sample size (*N* ≤ 200). The results for the 10-year graft survival rate indicated that, regardless of sample size, the graft survival rate in the PKD group was significantly higher than that in the non-PKD group (*N* > 200: OR 1.61, 95% CI: 1.53, 1.69; *N* = 14, I^2^ = 80.1%; *N* ≤ 200: OR 1.41, 95% CI: 1.01, 1.99; *N* = 6, I^2^ = 17.3%). In the analyses of the 1-year and 5-year graft survival rates, no significant associations were observed irrespective of sample size (see Table 2, Supplementary Figure S7). Subgroup analyses based on study design revealed that in retrospective cohort studies, the graft survival rate of the PKD group was significantly higher than that of the non-PKD group for the 10-year graft survival rate (OR 1.08, 95% CI: 0.86, 1.35; *N* = 14, I^2^ = 74.3%). Conversely, when combining the results of case-control studies, the better survival outcomes in the PKD group did not reach statistical significance (OR 1.83, 95% CI: 0.89, 3.77; *N* = 6, I^2^ = 44.3%). Furthermore, the results of the subgroup analyses for the 1-year and 5-year graft survival rates indicated no statistical significance in either retrospective studies or case-control studies (see Table 2, Supplementary Figure S8).

Subgroup analysis stratified by age (>45 years vs. ≤45 years) (Table 3) showed that among patients ≤45 years old, the PKD group demonstrated better graft survival rates at 1 year, 5 years, and 10 years compared to the non-PKD group; however, none of these differences reached statistical significance. Among patients >45 years old, the PKD group had superior 10-year graft survival compared to the non-PKD group (OR 1.63, 95% CI: 1.54–1.71, *N* = 13, I^2^=77.7%), but showed no significant differences in survival at 1 year or 5 years, with considerable heterogeneity present. Furthermore, subgroup analysis based on dialysis duration (>24 months vs. ≤24 months) (Table 3) revealed that regardless of dialysis duration, PKD patients had superior 10-year graft survival compared to non-PKD patients (>24 months: OR 1.89, 95% CI: 1.68–2.12, *N* = 7, I^2^=73.5%; ≤24 months: OR 1.47, 95% CI: 1.37–1.57, *N* = 6, I^2^=66.3%). However, there was no statistically significant difference in 1-year survival between the two groups. Conversely, among patients with shorter dialysis duration (≤24 months), the PKD group had inferior 5-year graft survival compared to the non-PKD group (OR 0.88, 95% CI: 0.83–0.94, *N* = 7, I^2^=80.5%), with considerable heterogeneity present.

### 3.4. Post-transplant complications

**Figure 4.**
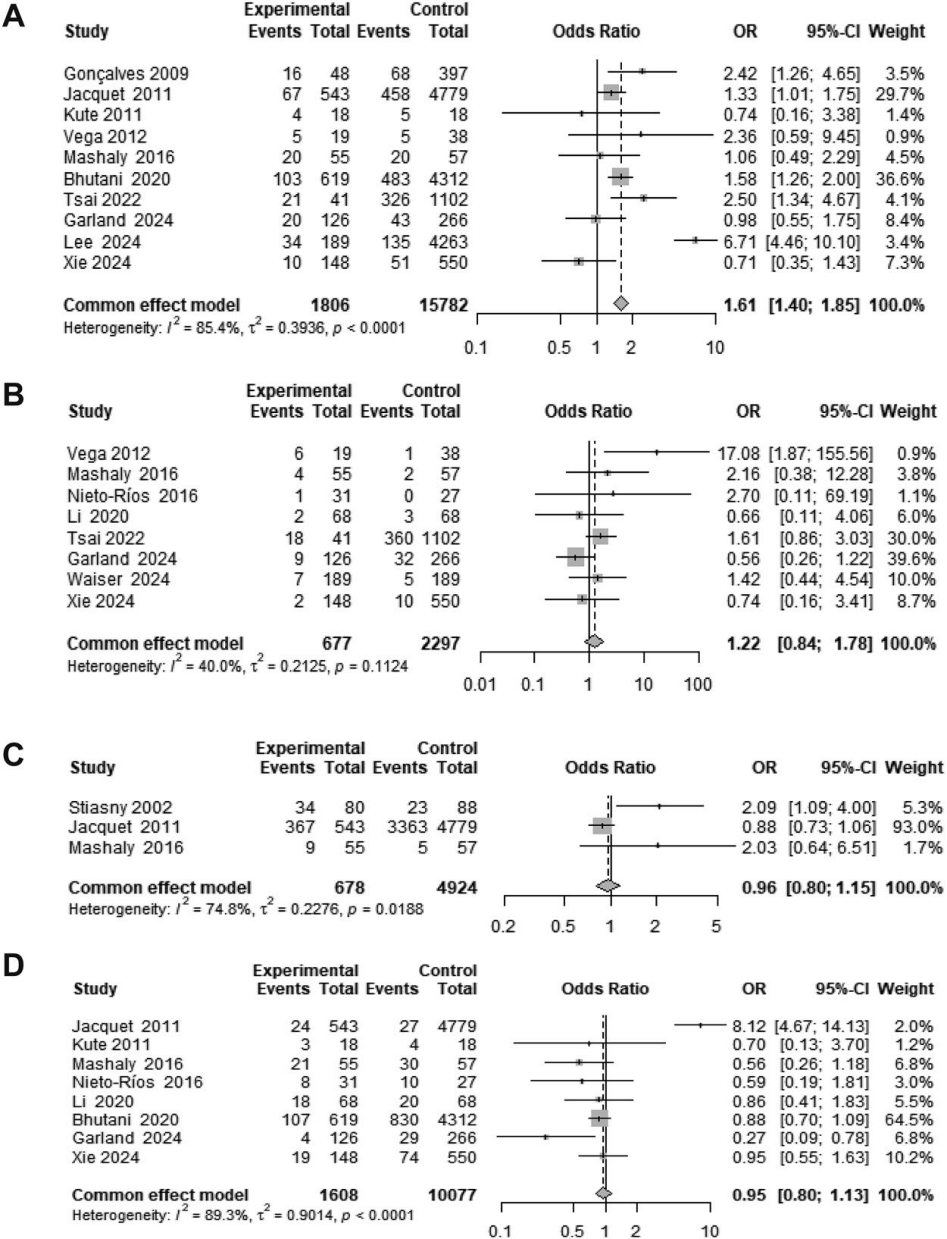
Forest plot of graft complications in the PKD group versus the non-PKD group. (a) post-transplant diabetes mellitus; (b) tumor; (c) infection; (d) acute rejection.

As illustrated in Figure 4, ten studies reported the incidence of PTDM. When these data were included in a meta-analysis, the incidence in the PKD group was found to be significantly higher than that in the non-PKD group (OR: 1.61; 95% CI: 1.40, 1.81; I^2^ = 85.4%; *p* < 0.05). Additionally, eight studies provided detailed data on malignancies. The meta-analysis results indicated that the incidence of malignancies was higher in the PKD group compared to the non-PKD group, although this difference did not reach statistical significance (OR: 1.22; 95% CI: 0.84, 1.78; I^2^ = 40.0%). Furthermore, data regarding infections and acute rejection were provided by three and eight studies, respectively. The results showed that the incidence of infections (OR: 0.96; 95% CI: 0.80, 1.15; I^2^ = 74.8%) and acute rejection (OR: 0.95; 95% CI: 0.80, 1.13; I^2^ = 89.3%) in the PKD group was lower than that in the non-PKD group; however, these results did not attain statistical significance.

### 3.5. Quality assessment

**Figure 5.**
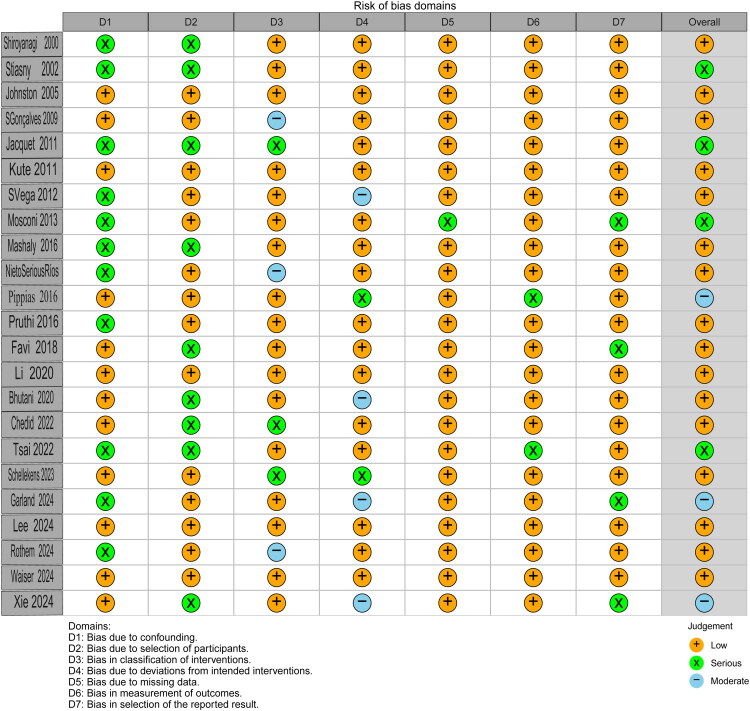
Quality assessment of the included studies.

The quality assessment results based on the ROBINS-I tool (see Figure 5) indicated that the studies were rated as having a moderate risk of bias due to the influence of confounding factors. Among these, six studies were multicenter retrospective studies, while the others were single-center retrospective studies. TORee studies did not report patient survival rates, and an additional two studies did not provide graft survival rates. Only five studies adjusted for age, sex, dialysis modality prior to transplantation, and comorbidities, while the remaining studies failed to adjust for significant confounding factors that could impact outcomes, including age, sex, donor type, diabetes, cardiovascular disease, and immunosuppressive regimens.

## 4. Discussion

The results indicate that, compared to non-PKD patients, PKD patients exhibit advantages in short-term survival rates, while significant benefits in graft survival rates are observed during long-term follow-up. Additionally, subgroup analyses explored the impact of donor type, sample size, and study design on PKD kidney transplant patients, revealing particularly favorable trends in survival and graft survival rates among PKD patients in retrospective studies and those with unspecified donor sources. We also found that the incidence of PTDM was higher in the PKD group compared to the non-PKD group; however, no significant differences were noted regarding acute rejection, infections, or malignancies.

PKD patients demonstrated a significantly higher 1-year survival rate following kidney transplantation compared to non-PKD patients (OR 1.3, 95% CI: 1.08–1.56; I^2^ = 0%), although no significant differences were observed in long-term survival rates. This may be attributed to the fact that, despite the progressive replacement of renal parenchyma by cysts in PKD patients, some residual renal units may retain their erythropoietin (EPO) secretion function [20]. In contrast, other ESRD patients, such as those with diabetic nephropathy, hypertensive nephropathy, and chronic glomerulonephritis, completely lose their ability to produce EPO [21]. Anemia can lead to a compensatory high-output state of the heart, increasing myocardial oxygen consumption and thereby elevating the risk of early cardiovascular events in kidney transplant recipients. Correcting anemia and improving hemoglobin levels can alleviate this burden [22,23]. Furthermore, the study by Rothem et al. confirmed that elevated hemoglobin levels are associated with improved estimated glomerular filtration rates 1-year post-transplant, contributing to higher graft survival rates among PKD patients [24]. Over time, as functional renal tissue in PKD patients further diminishes and EPO production decreases [20], the transplanted kidney gradually becomes the primary source of EPO, reducing the differences in anemia between PKD and non-PKD patients.

In addition to the advantages related to anemia, cardiovascular health and metabolic factors also significantly influence the post-transplant survival rate of PKD patients. First, as a genetic disorder distinct from diabetic or hypertensive neporopathy, PKD patients typically reach ESRD at a younger age with lower prevalences of diabetes and preexisting cardiovascular disease at transplantation [25]. This favorable cardiovascular risk profile reduces perioperative and early post-transplant cardiovascular events (e.g., myocardial infarction, stroke), directly enhancing 1-year survival [26]. Second, the significantly lower prevalence of diabetes in PKD recipients mitigates diabetes-associated complications including cardiovascular disease, retinopathy, and ketoacidosis [12]. Critically, pre-transplant diabetes confers *a* > 3-fold higher risk of post-transplant cardiovascular events (38.1% [95% CI: 32.1%-45.3%]) versus non-diabetic recipients (11.8% [95% CI: 9.2%-15.0%]) [27].

Moreover, PKD patients are more susceptible to complications such as hypertension, diabetes, polycystic liver disease, and intracranial aneurysms [9,28–31], which may counterbalance their early survival advantages. The research by Li et al. also confirmed that there were no significant differences in long-term survival rates, pneumonia, cardiovascular events, or malignancy risks between PKD recipients and the control group^32^, which aligns with our findings.

In terms of graft survival rates, PKD patients exhibited a significantly higher 10-year graft survival rate compared to the non-PKD group (OR 1.60; 95% CI: 1.52–1.62), while no significant differences were noted in the short term (1-year and 5-year). Patients with PKD rarely experience recurrence of their primary disease (PKD) in the transplanted kidney. In contrast, other primary diseases, such as focal segmental glomerulosclerosis (FSGS), membranous glomerulopathy, or diabetic nephropathy, show low recurrence rates within the 1-year post-transplant, with a 5-year recurrence rate of primary diseases ranging from 2.8% to 15%, more than 50% of which are attributed to the recurrence of IgA nephropathy. The recurrence of primary kidney diseases is significantly associated with graft failure [26,33,34]. Furthermore, some PKD patients underwent nephrectomy for polycystic kidneys before, during, or after kidney transplantation, which effectively addressed their primary disease and extrarenal complications, thereby enhancing graft survival rates [35,36]. These factors contribute to the lack of significant differences in short-term graft survival rates among PKD patients, while their long-term graft survival rates surpass those of non-PKD patients.

In the context of superior graft survival rates for patients, no significant differences were observed between studies involving deceased donors and those involving living donors, while groups with unspecified donor sources demonstrated better graft survival rates. This may be partly attributable to a higher proportion of living donor kidneys included in studies with unspecified donor types. Living donor kidneys typically come from healthy individuals, potentially creating a more favorable immunological environment that promotes graft acceptance and improves outcomes [37]. Conversely, organs from deceased donors are often damaged due to brain death or circulatory failure, resulting in variable organ quality [38,39]. Therefore, future research should focus on analyzing donor types to more precisely explore the relationships among donor characteristics, graft quality, and long-term survival patterns, thereby providing more valuable insights.

Our findings indicate that only retrospective studies demonstrate that PKD patients have better short-term (1-year) survival rates and long-term (10-year) graft survival rates compared to non-PKD patients, while case-control studies show no association between the presence of PKD and patient or graft survival rates. This discrepancy, observed when comparing different study designs, can typically be attributed to the strengths and limitations inherent in each design. In retrospective studies, a significant association exists between PKD and kidney transplant outcomes. However, this association may be influenced by the quality of historical data, differences among data collectors, causal relationships between exposures and outcomes, and unmeasured confounding factors, leading to variations in results. Conversely, in case-control studies, researchers may encounter challenges such as recall bias, selection bias, and difficulties in matching factors with the control group. Additionally, retrospective studies often rely on large sample registry databases (e.g., UNOS), which provide strong statistical power and facilitate the detection of small differences; in contrast, case-control studies tend to have smaller sample sizes, particularly when the outcome incidence is low, potentially resulting in false-negative findings.

Our study also found that the incidence of PTDM was higher in the PKD group compared to the non-PKD group, consistent with previous research [29,40,41]. The pathogenesis of PKD involves multiple genes, with the most common mutations being in PKD1 and PKD2, which encode polycystin-1 and polycystin-2, respectively [42]. These polycystins play an essential role in maintaining glucose homeostasis outside of the kidneys and in other organs such as the pancreas, liver, and skeletal muscle [43], and may be closely related to poor glycemic control. Studies have shown that PKD1 mutations can enhance glycolytic processes in the kidneys and cells of PKD patients and animal models. Research by Rowe et al. demonstrated that glucose utilization in renal cells of PKD1-/- mice shifted toward increased lactate production, supporting the role of ciliary components in cellular glucose metabolism [44]. In addition to genetic mutations, other significant contributors to the development and progression of PTDM include immunosuppressive agents (specifically calcineurin and mTOR inhibitors), viral infections, family history of diabetes, chronic corticosteroid use, deceased donor status, obesity, metabolic syndrome, and proteinuria [42,45–47]. Furthermore, in the analysis of the incidence of acute rejection, infections, and malignancies between the two groups, no significant differences were found. This may be due to the limited number of studies addressing these complications, particularly as only three studies provided relevant data on the incidence of infections. Consequently, robust conclusions cannot yet be drawn, and further research is needed to confirm these findings.

Our study also has several limitations. First, the studies included in this meta-analysis involved patient populations with varying comorbidities, immunosuppressive regimens, and demographic characteristics. PKD itself is a genetic disease often associated with extrarenal manifestations, such as hepatic cysts and intracranial aneurysms. The differences in patient characteristics across studies may introduce confounding factors that could affect the pooled outcomes. Second, there is limited data on donor types, and the underlying reasons for the superior survival and graft survival rates in the groups without specified donor sources remain unclear. We hope that future multi-center studies can achieve a more homogeneous cohort and include detailed donor information to enhance the aggregation of results and provide more reliable evidence. Third, subgroup analyses regarding whether PKD patients underwent PKD surgery prior to kidney transplantation were not conducted, which may impact the study results. Fourth, we did not search for gray literature, which could minimize the potential for publication bias. Fifth, since most of the included studies were retrospective cohort studies, we could not entirely eliminate the influence of confounding factors. Additionally, the number of case-control studies was small, and sample sizes were limited, which may lead to shortcomings in the long-term follow-up data. Sixth, although polycystic liver disease and intracranial aneurysms are important extrarenal manifestations of ADPKD with potential survival implications, the pervasive lack of data in primary studies precluded assessment of their potential confounding effect on the survival differences observed in this meta-analysis. Finally, PKD patients typically require immunosuppressive therapy prior to kidney transplantation to manage PKD, and these factors may also influence the outcomes.

## 5. Conclusion

This systematic review is the first to report that PKD kidney transplant recipients have better short-term survival rates and long-term graft survival rates compared to non-PKD patients; conversely, there are no differences in long-term survival rates and short-term graft survival rates. Additionally, a high incidence of PTDM was observed among PKD patients. These findings provide new insights for transplant physicians, highlighting the importance of implementing personalized management and monitoring of blood glucose levels for PKD patients after kidney transplantation, which is crucial for improving long-term prognosis and reducing complications in this patient population. Furthermore, future studies should be conducted with large sample sizes, multi-center involvement, and long-term follow-up periods to eliminate the influence of confounding factors such as study methodology, donor type, and sample size, thereby further validating our conclusions.

## Supplementary Material

Supplemental Material

Supplementary_Material_Search_Strategy.docx

## Data Availability

The datasets generated during and/or analyzed during the current study are available from the corresponding author on reasonable request.
