# Supplementary material for: Impact of polycystic kidney disease on outcomes after renal transplantation: systematic review and meta-analysis
Source: Ren Fail. 2026 Jan 19;47(1):2611618. doi: 10.1080/0886022X.2025.2611618 (PMC12818323; doi:10.1080/0886022X.2025.2611618)

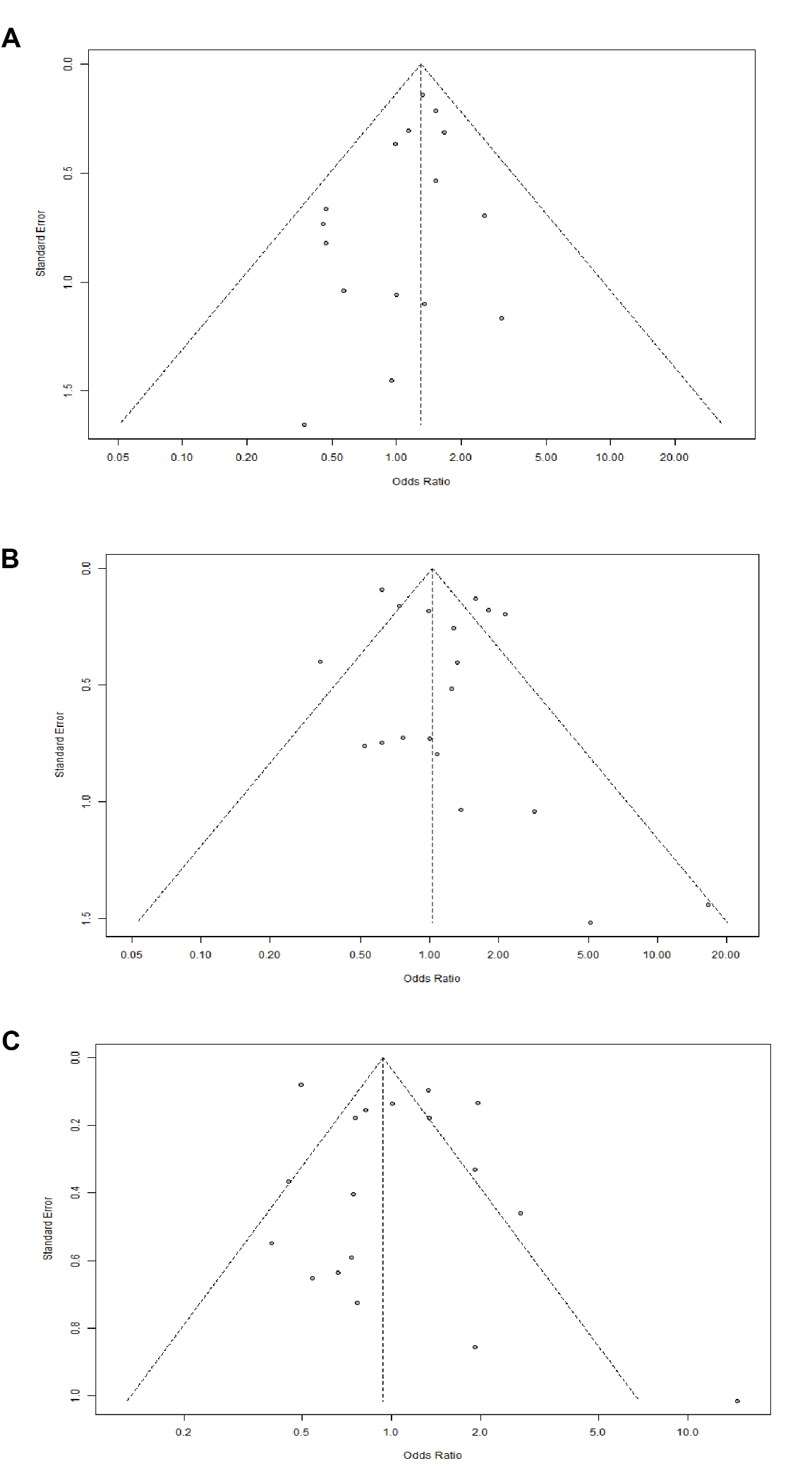


Supplement Figure 1. Funnel plot comparing the survival rates after kidney transplantation between PKD and non-PKD patients. (A) 1-year survival rate; (B) 5-year survival rate; (C) 10-year survival rate.


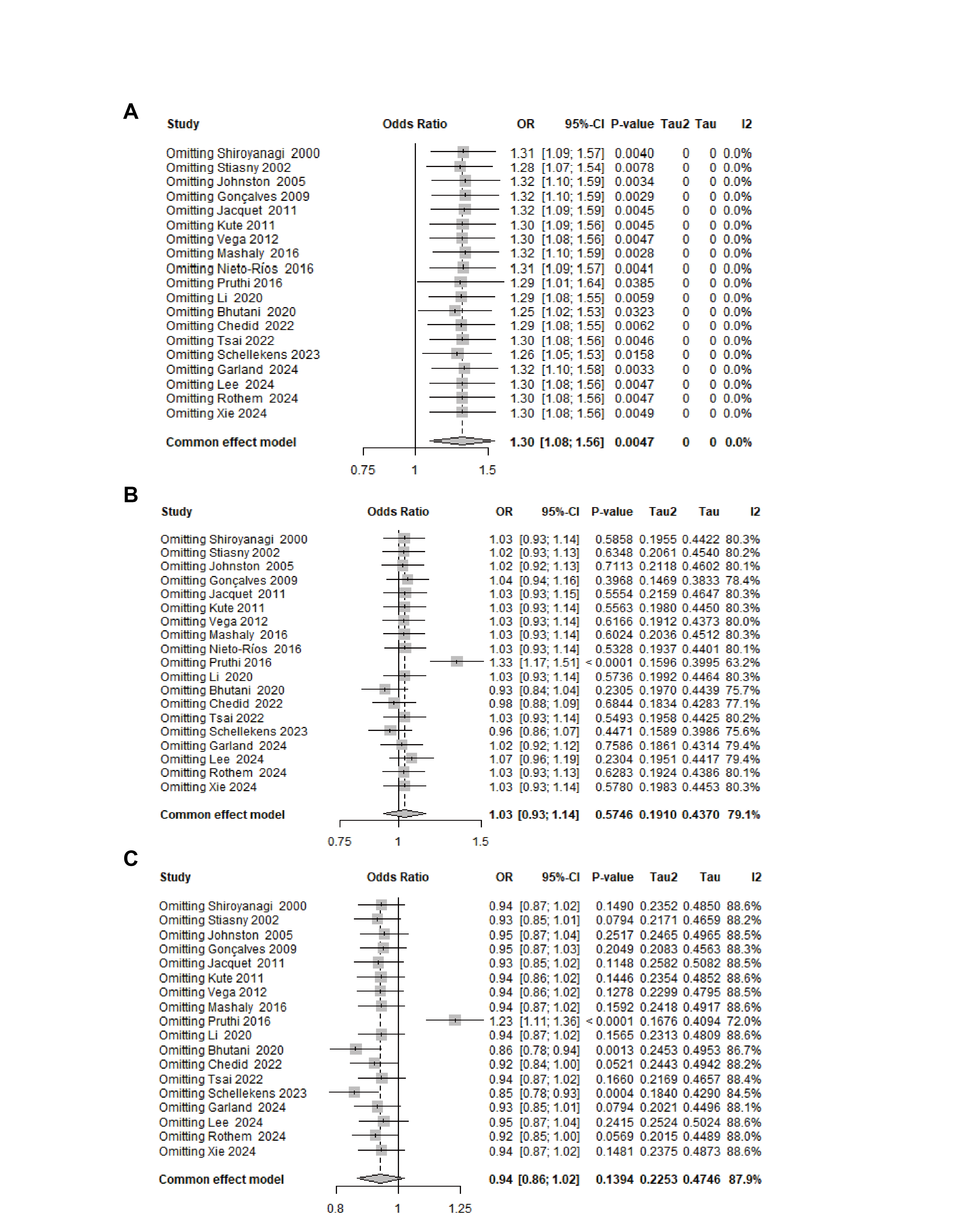


Supplement Figure 2. Sensitivity analysis comparing the survival rates after kidney transplantation between PKD and non-PKD patients. (A) 1-year survival rate; (B) 5-year survival rate; (C) 10-year survival rate.


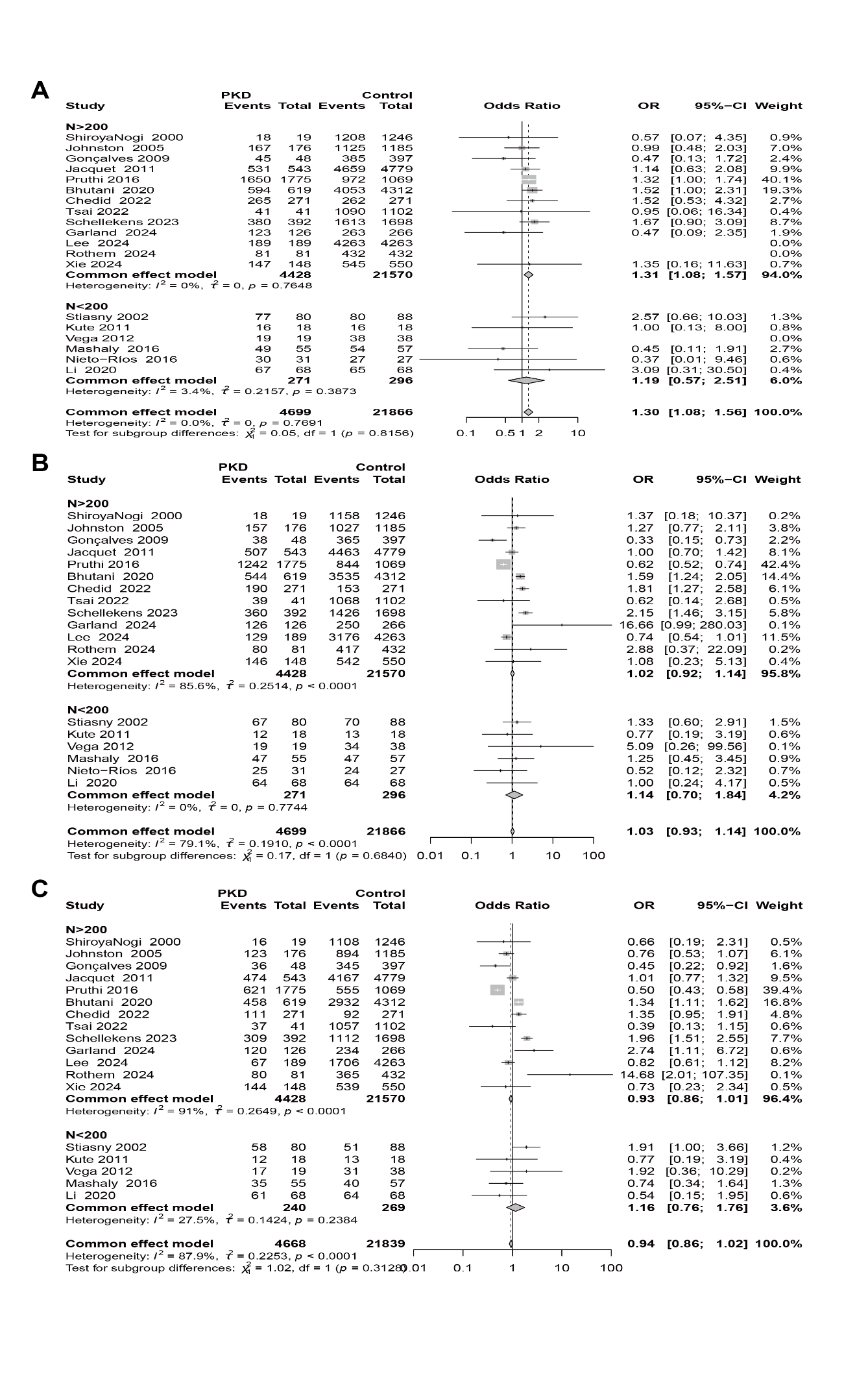


Supplement Figure 3. Forest plot of subgroup analysis comparing the survival rates after kidney transplantation between PKD and non-PKD patients based on sample size (N > 200, N ≤ 200). (A)
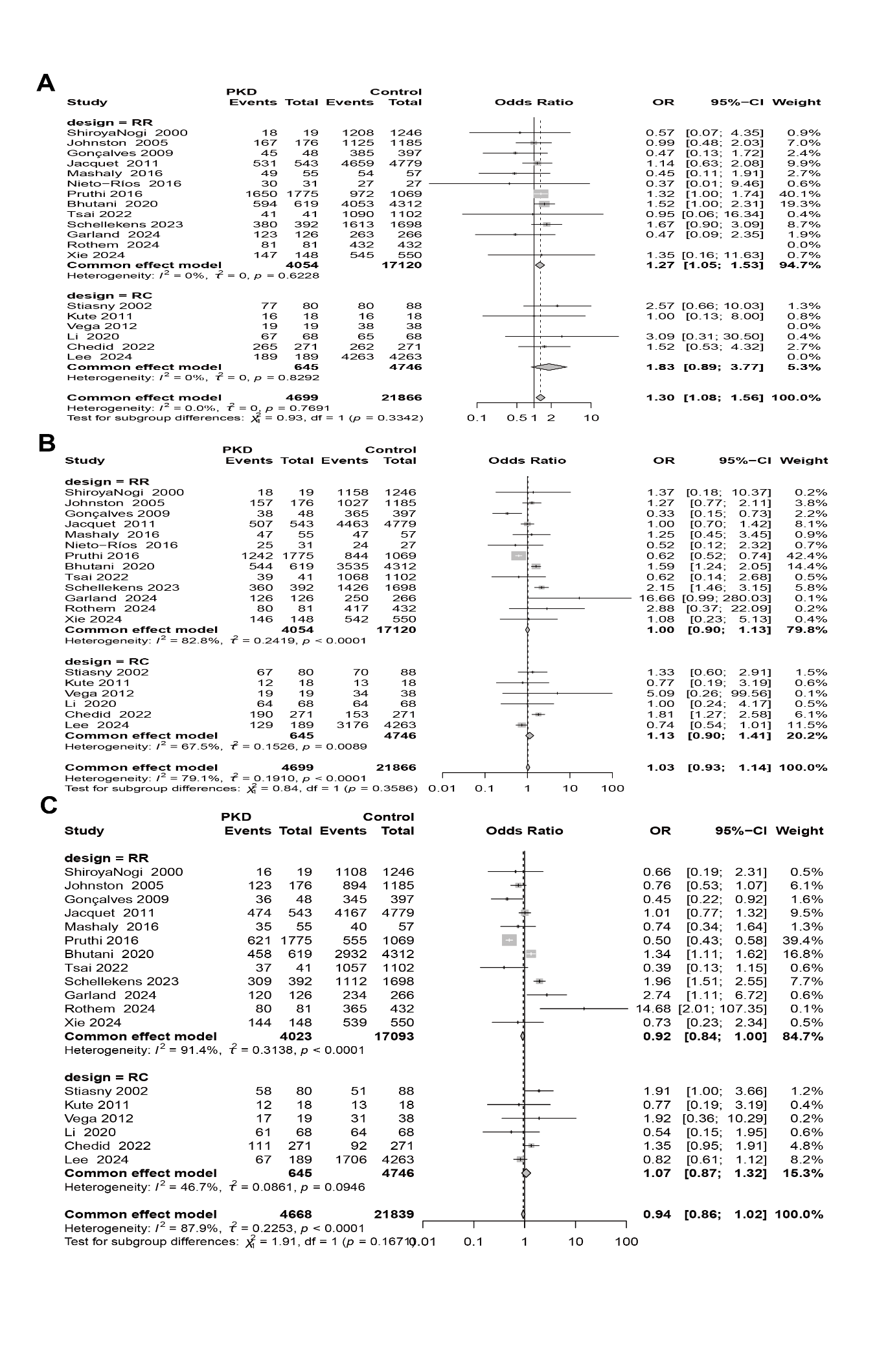
1-year survival rate; (B) 5-year survival rate; (C) 10-year survival rate.

Supplement Figure 4. Forest plot of subgroup analysis comparing the survival rates after kidney transplantation between PKD and non-PKD patients based on study type. (A) 1-year survival rate; (B) 5-year survival rate; (C) 10-year survival rate. RR: retrospective studies; RC: case-control studies.


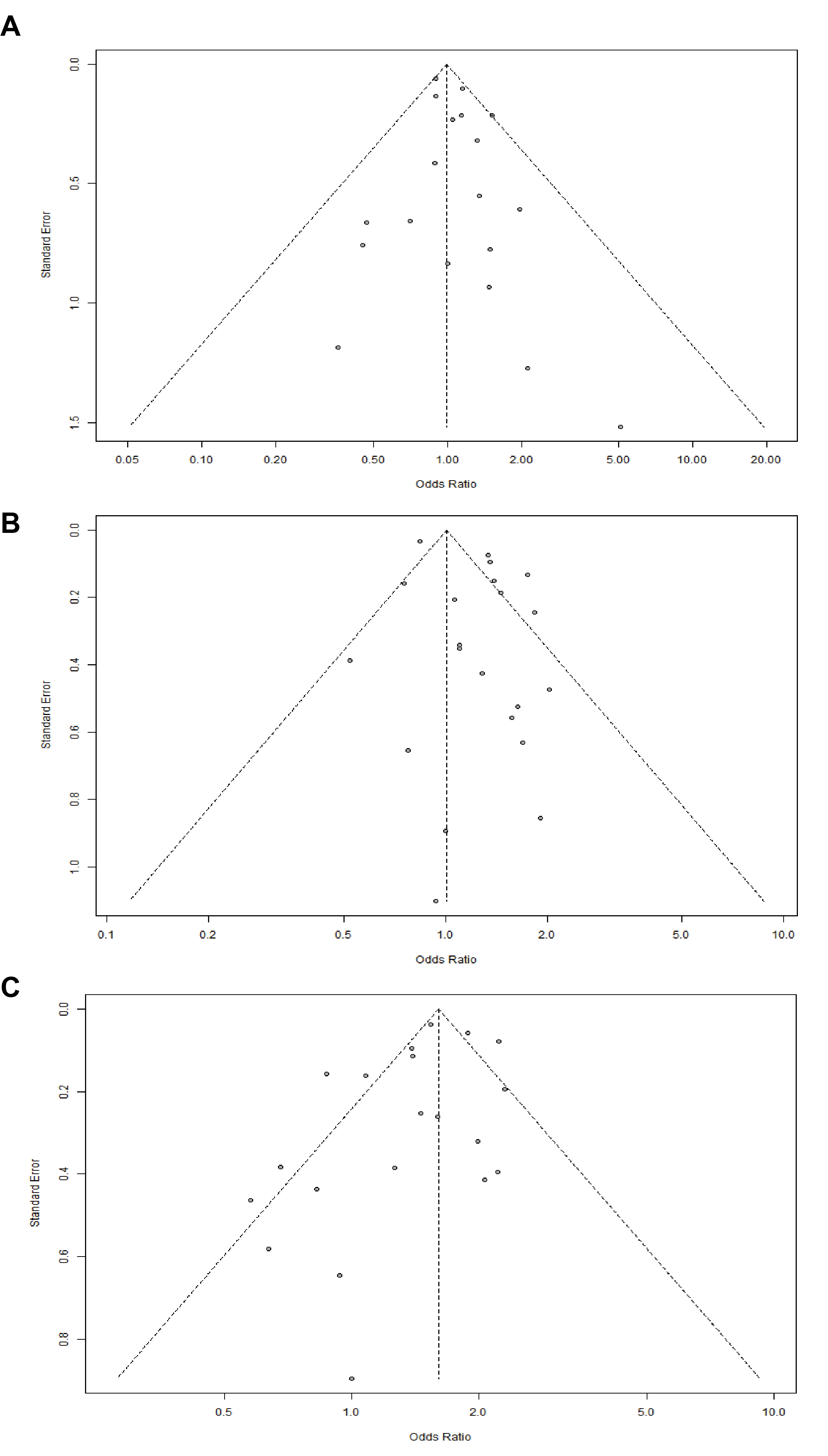
 Supplement Figure 5. Funnel plot comparing the graft survival rates after kidney transplantation between PKD and non-PKD patients. (A) 1-year graft survival rate; (B) 5-year graft survival rate; (C) 10-year graft survival rate.


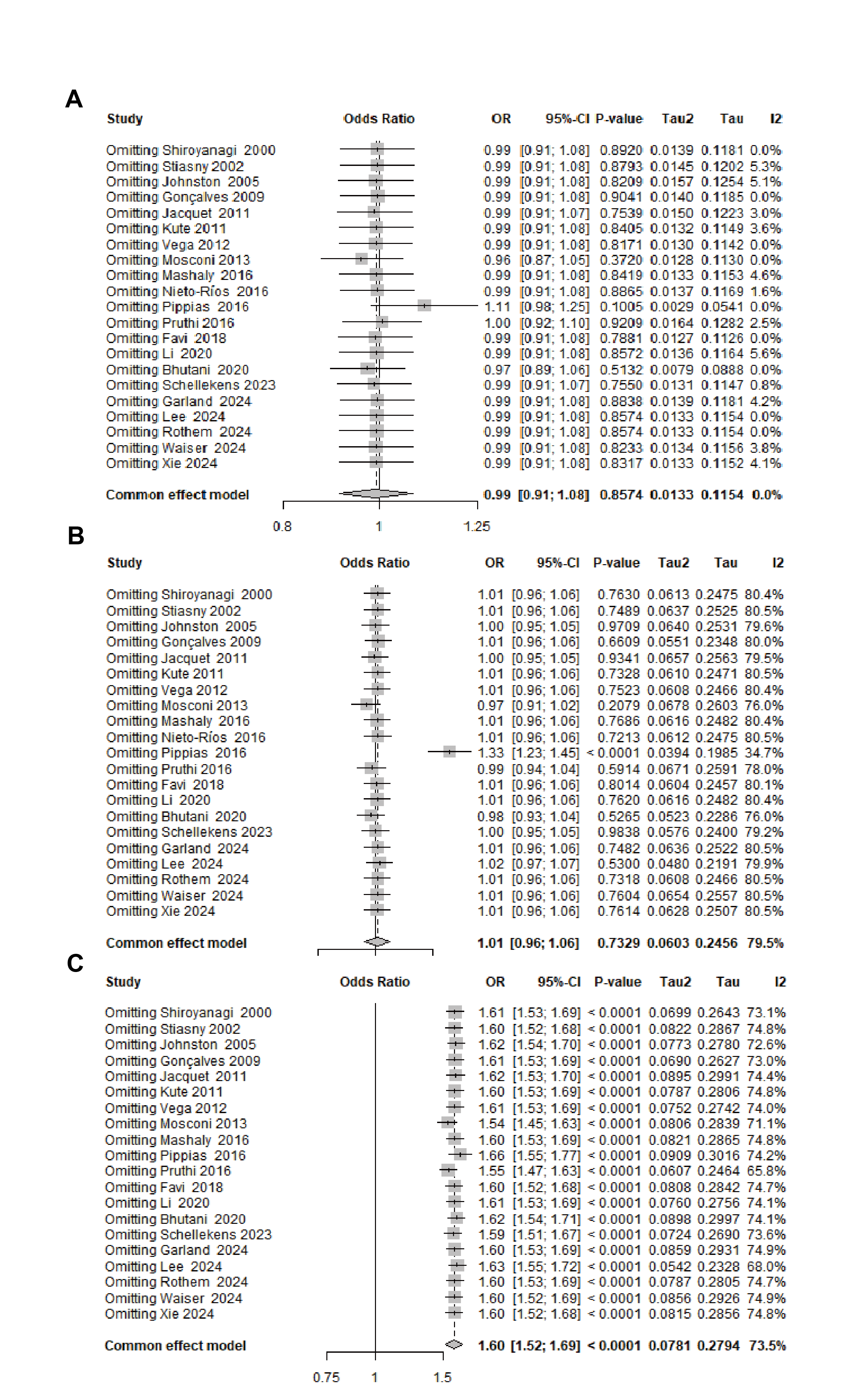
Supplement Figure 6. Sensitivity analysis comparing the graft survival rates after kidney transplantation between PKD and non-PKD patients. (A) 1-year graft survival rate; (B) 5-year graft survival rate; (C) 10-year graft survival rate.


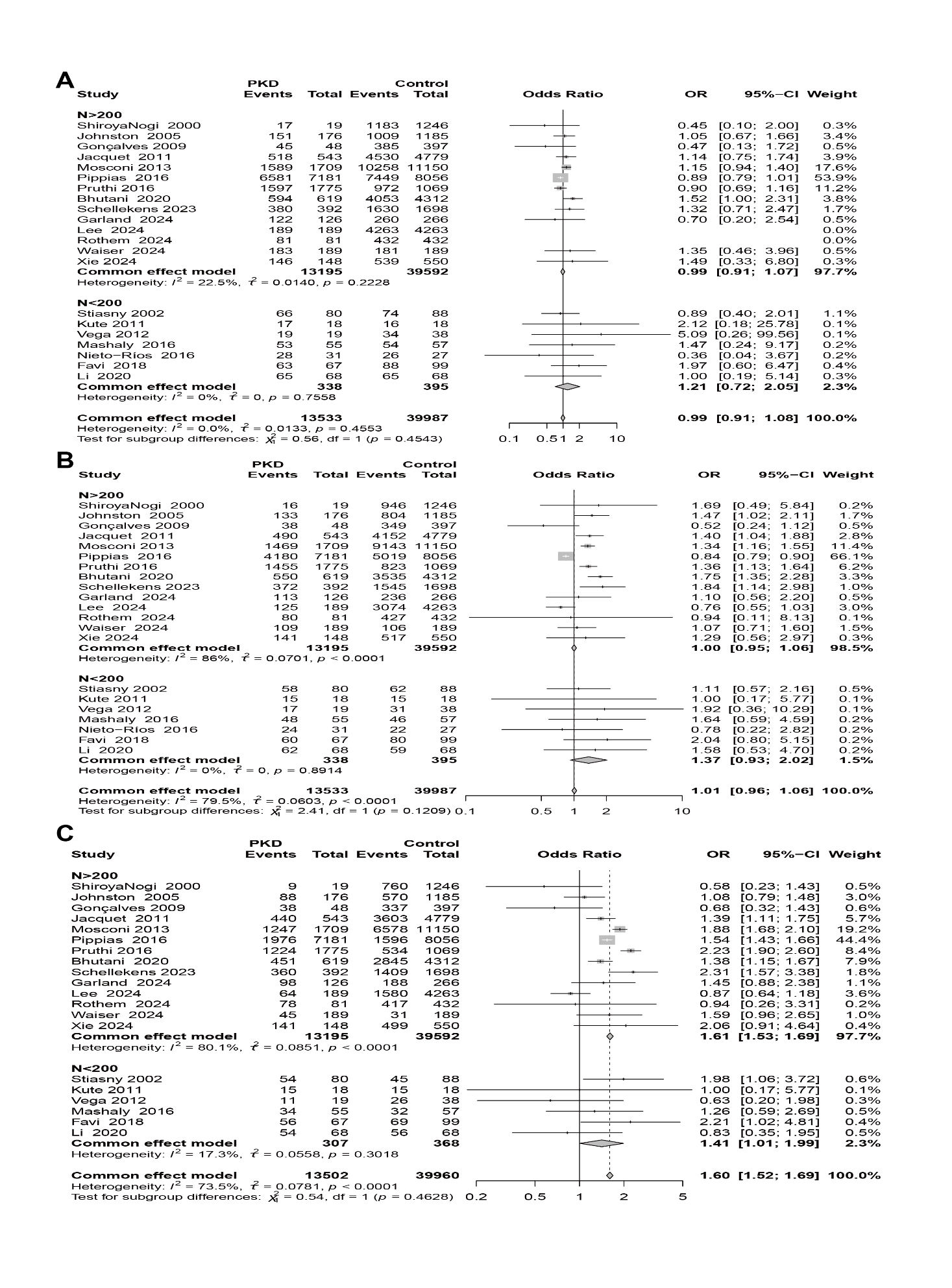


Supplement Figure 7. Forest plot of subgroup analysis comparing the graft survival rates after kidney transplantation between PKD and non-PKD patients based on sample size (N > 200, N ≤ 200). (A) 1-year graft survival rate; (B) 5-year graft survival rate; (C) 10-year graft survival rate.

Supplement Figure 8. Forest plot of subgroup analysis comparing the graft survival rates after kidney transplantation between PKD and non-PKD patients based on study type. (A) 1-year graft survival rate; (B) 5-year graft survival rate; (C) 10-year graft survival rate. RR: retrospective studies; RC: case-control studies.
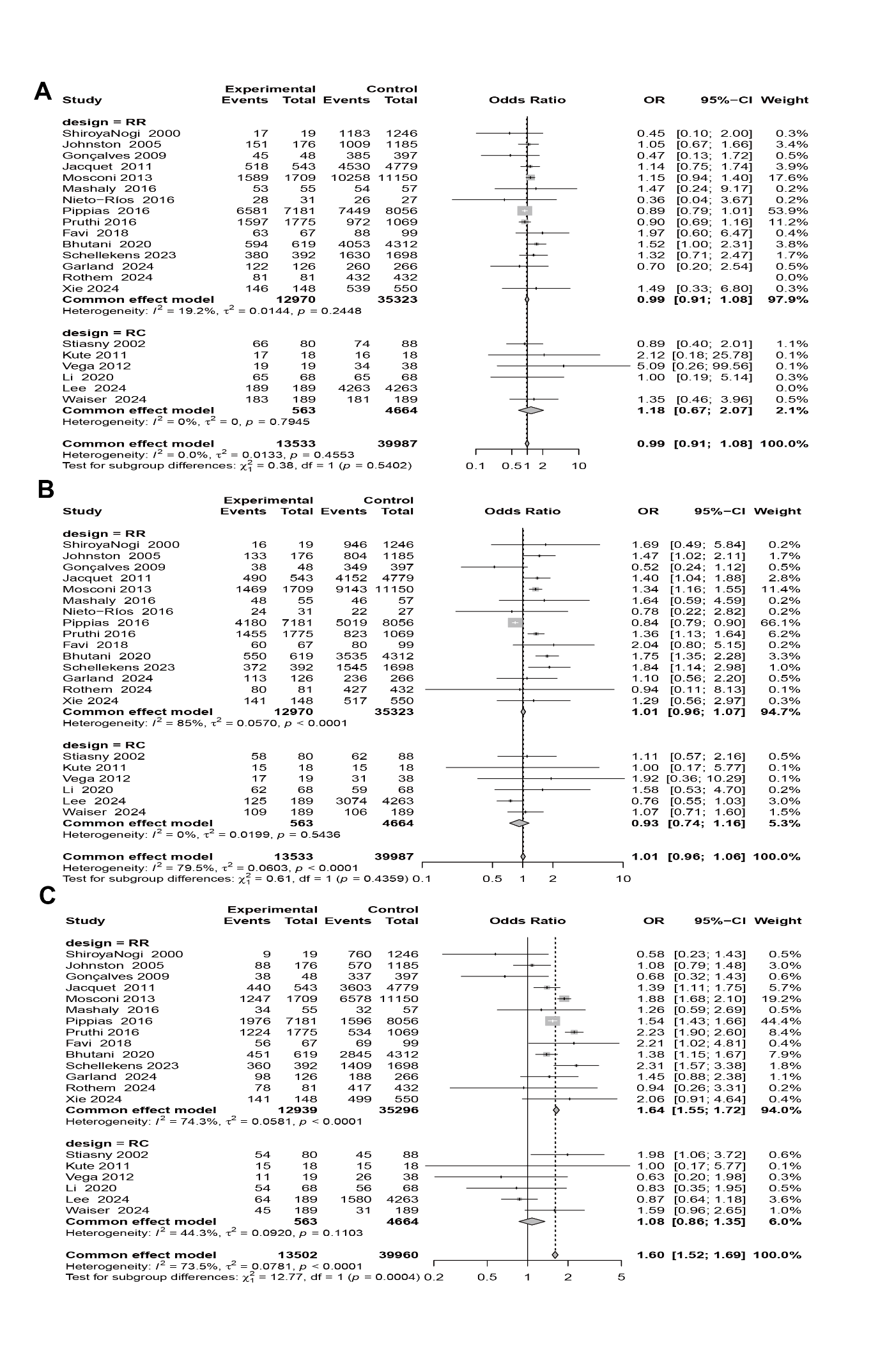

Supplement: Supplemental Material [file IRNF_A_2611618_SM0726.docx]
