# Supplementary material for: Impact of polycystic kidney disease on outcomes after renal transplantation: systematic review and meta-analysis
Source: Ren Fail. 2026 Jan 19;47(1):2611618. doi: 10.1080/0886022X.2025.2611618 (PMC12818323; doi:10.1080/0886022X.2025.2611618)
Supplement: Supplementary_Material_Search_Strategy.docx [file IRNF_A_2611618_SM8917.docx]

Supplementary table 1. Search strategy used.

| A) PubMed |
| --- |
| #1 (Polycystic Kidney Diseases OR PKD OR Autosomal Dominant Polycystic Kidney OR ADPKD OR Polycystic)  #2 (renal transplant OR kidney transplant)  #3 (clinical outcomes OR patient survival OR graft survival OR overall survival)  #4 (#1 AND #2 AND #3)  #5 (Autobiography[ptyp] OR Bibliography[ptyp] OR Biography[ptyp] OR pubmed books[filter] OR Case Reports[ptyp] OR Consensus Development Conference[ptyp] OR Directory[ptyp] OR Duplicate Publication[ptyp] OR Editorial[ptyp] OR Systematic Reviews OR Meta Analysis OR Review[ptyp] OR Festschrift[ptyp] OR Guideline[ptyp] OR Interview[ptyp] OR News[ptyp] OR Newspaper Article[ptyp] OR Retracted Publication[ptyp] OR Twin Study[ptyp] OR Video-Audio Media[ptyp])  #6 (#4 NOT #5) |
| B) Embase |
| #1 'clinical outcomes' OR 'patient survival'/exp OR 'graft survival'/exp OR 'overall survival'  #2'renal transplant'/exp OR 'kidney transplant'  #3'polycystic kidney diseases'/exp OR 'pkd' OR 'autosomal dominant polycystic kidney'/exp OR 'adpkd' OR 'polycystic'  #1 AND #2 AND #3 |
| C) Scopus |
| #1 TITLE-ABS-KEY("Polycystic Kidney Diseases" OR PKD OR "Autosomal Dominant Polycystic Kidney" OR ADPKD OR Polycystic)  #2 TITLE-ABS-KEY("renal transplant" OR "kidney transplant")  #3 TITLE-ABS-KEY("clinical outcomes" OR "patient survival" OR "graft survival" OR "overall survival")  #1 and #2 and #3 |
